# Pulmonary influenza A virus infection leads to suppression of the innate immune response to dermal injury

**DOI:** 10.1371/journal.ppat.1007212

**Published:** 2018-08-23

**Authors:** Meredith J. Crane, Yun Xu, William L. Henry, Sean P. Gillis, Jorge E. Albina, Amanda M. Jamieson

**Affiliations:** 1 Division of Biology and Medicine, Department of Molecular Microbiology and Immunology, Brown University, Providence, Rhode Island, United States of America; 2 Division of Biology and Medicine, Department of Molecular Biology, Cell Biology, and Biochemistry, Brown University, Providence, Rhode Island, United States of America; 3 Department of Surgery, Rhode Island Hospital and the Warren Alpert School of Medicine of Brown University, Providence, Rhode Island, United States of America; St. Jude Children's Research Hospital, UNITED STATES

## Abstract

The innate immune system is responsible for many important functions in the body including responding to infection, clearing cancerous cells, healing wounds, and removing foreign substances. Although many of these functions happen simultaneously in life, most laboratory studies of the innate immune response focus on one activity. How the innate immune system responds to concurrent insults in different parts of the body is not well understood. This study explores the impact of a lung infection on the cutaneous wound healing process. We used two complimentary models of injury: the excisional tail wound and subcutaneous implantation of polyvinyl alcohol (PVA) sponges. These models allow for assessment of the rate of closure and measurement of cellular and cytokine responses during acute wound healing, respectively. When mice with these healing wounds were infected with influenza A virus (IAV) in the lung there was a delay in wound healing. The viral lung infection suppressed the innate immune response in a healing wound, including cellular infiltrate, chemokines, growth factors, and cytokines. However, there was not a global immune suppression as there was an increase in inflammation systemically in mice with both infection and healing wounds compared to mice with only wounds or IAV infection. In addition, the lung immune response was largely unaffected indicating that responding to a lung infection is prioritized over a healing wound. This study introduces the concept of immune triage, in that when faced with multiple insults the immune system prioritizes responses. This paradigm likely applies to many situations that involve the innate immune system, and understanding how the innate immune system handles multiple insults is essential to understanding how it can efficiently clear pathogens while responding to other inflammatory events.

## Author summary

In a natural setting, the innate immune system is frequently faced with multiple insults, against which it must mount overlapping inflammatory responses. We are interested in how the innate immune system deals with multiple, simultaneously occurring inflammatory insults, and if the response to one will take priority. For example, the innate immune system is essential in mediating both the early control of pathogen replication in infected tissue and in the early stages of wound healing. Pulmonary infections occur frequently in injured patient populations; therefore, we set out to determine the impact of a respiratory infection on a healing wound. To examine this, mice with healing dermal wounds were infected with influenza A virus (IAV), a common cause of viral pneumonia. We found that the innate immune response to the lung infection took priority at the expense of the healing wound, in that the initial control of viral replication in the lung was not impacted, while the wound healing response was suppressed. Very little work has been done examining how the immune response can respond to overlapping inflammatory insults. Our work shows that not all immune responses are created equal, and that the cells of the innate immune system are preferentially routed towards fighting a lung infection rather than the healing dermal wound. This apparent prioritization of the innate immune response opens up a new direction of study. It is relevant to many fields where competing insults may alter the disease state.

## Introduction

The immune system plays roles in multiple processes in the body including the response to infection, tissue repair, development, cancer immunosurveillance, and maintenance of homeostasis [1–4]. Dysregulation of these processes can lead to a disease state [5–8]. Most studies of the immune response focus on just one of the functions of the immune system. In reality, however, the immune system, especially the innate immune system, must be able to respond to multiple insults at the same time. Understanding how the innate immune system is equipped to handle simultaneous and disparate inflammatory events will provide greater insight into the complexity of the immune response. While some progress has been made in understanding how the innate immune system is altered when faced with multiple infections [9–16], how the onset of an infection alters the immune response to an ongoing non-infectious insult, such as a cutaneous wound, has not been well explored.

Wound healing is an essential process in human health, and its proper progression is critical to a full recovery from trauma and surgery. Innate immunity plays an important role in initiating wound repair. Neutrophils and monocytes infiltrate the wound from the periphery based on signaling from chemokines, cytokines, and interactions with adhesion molecules on the activated endothelium [2,17–22]. The earliest phase of cutaneous wound repair is characterized by an inflammatory cytokine milieu, including TNF-α, IL-6, IL-1α, IL-1β, and IFN-γ, as well as leukocyte-attracting chemokines such as CXCL1, CXCL5, CXCL10, CCL2, CCL3, CCL4, and CCL5 [18,19,23–28]. Neutrophils and inflammatory monocytes are rapidly attracted to wounds following the release of DAMPs and chemokines. [2,18–20,24,28]. Together, these earliest-responding cells aid in the clearance of damaged tissue and coordinate downstream responses required for tissue repair, and disruption of this acute phase negatively impacts downstream healing. Once in the wound, monocytes differentiate into repair macrophages; as a consequence, impairment of monocyte trafficking to wounds through loss of CCL2 or CCL3 signaling disrupts angiogenic and fibrotic responses required for proper wound healing [3, 29–31]. Similarly, blockade of neutrophil trafficking after myocardial infarction has been shown to impair cardiac repair [32]. Efferocytic clearance of apoptotic neutrophils by wound macrophages dampens inflammatory responses and promotes the transition to a repair phenotype, and loss of this interaction can prolong the inflammatory phase of wound healing [23, 32–34].

In addition to the complications that can arise from impaired wound healing, many co-morbidities, including infection of the wound, metabolic disorders, increased age and other environmental and genetic factors, can delay wound healing [8,17,21, 35–37]. There is also evidence that systemic disorders, such as sepsis, suppress wound healing [38,39]; however, sepsis is an extreme condition that causes widespread changes in physiology including decreased oxygenation and altered immune responses [38]. One area that is understudied is the effect of a distal infection, such as pneumonia, on the dermal wound healing process.

Pulmonary infections frequently occur after injury or trauma, and patients with infections have longer hospital stays and increased morbidity and mortality [40,41]. A recent report provides a link between the influenza season and increased risk of complications after surgery [42]. While it is generally thought that trauma impacts the ability to respond to a pulmonary infection [43], there are fewer studies that investigate the impact that lung infections, in particular viral infections, have on the wound healing response. Those that do exist mainly focus on viral infections of the wound site itself [44,45]. It remains an open question how a viral lung infection that is contained in one region of the body impacts the host’s ability to respond to tissue damage in a distinct region.

The acute cellular and cytokine responses to pulmonary influenza infection and dermal wounds share many common features. Recruitment and activation of innate immune cells such as neutrophils and monocytes by DAMPs, PAMPs, cytokines, and chemokines, are essential for the early control of pulmonary viral infection [21, 22, 27, 46]. Depletion of neutrophils and monocytes during influenza infection is linked to increased viral replication, although the accumulation of inflammatory monocytes after infection has also been shown to contribute to lung injury [27, 28, 46–50]. Given the overlap in innate immune cells and factors that are important in both wound healing and the early control of viral infection, we hypothesized that initiation of a pulmonary viral infection would disrupt the innate immune response at a distal cutaneous wound.

To explore the interconnectedness of the innate immune response when simultaneously responding to infection and injury, we used a viral lung infection model combined with two complementary murine wounding models. The first model is an excisional skin wound to the tail that allows for assessment of the rate of wound closure. The second model, subcutaneous implantation of polyvinyl alcohol (PVA) sponges, allows for the measurement of cellular and cytokine responses during acute wound healing. Wounded mice were subsequently infected with influenza A virus (IAV), which remains confined to the lung and does not spread systemically. Excisional skin wounds experienced delayed closure in mice that also had an IAV pulmonary infection. Examination of acute healing responses using the sponge implantation model revealed that wounds from mice with a pulmonary IAV infection had reduced cellularity and cytokine concentrations and a resulting decrease in the reparative growth factor, VEGF-A. These data demonstrate that the presence of a pulmonary infection disrupts the inflammatory phase of wound healing by altering the innate immune responses that drive the initial stages of repair. This impairment was not a result of systemic immunosuppression, as circulating cytokine and cellular responses remained intact. Investigating the interconnectedness of the early cellular and cytokine responses to these concurrent insults is paramount to a complete understanding of the immune response. This new area of study has exciting implications in the field of innate immunity, and has relevance for many disease models. How immune responses to infection, injury, development, or cancer influence each other and the ability to maintain a healthy organism is an important new area of future work.

## Results

### Infection with IAV delays cutaneous wound healing

For all experiments, mice were first wounded by tail skin excision or PVA sponge implantation, and infected with IAV 24 hours later. The treatments were timed in this way to mimic the onset of these inflammatory events in the clinical setting. Wounding by PVA sponge implantation causes measureable systemic inflammation and depletion of inflammatory monocytes from the circulation as they marginate to the wound within 24 hours [19]. IAV was administered at this time point to maximize the overlap of systemic responses from wounding and pulmonary infection.

**Fig 1:**
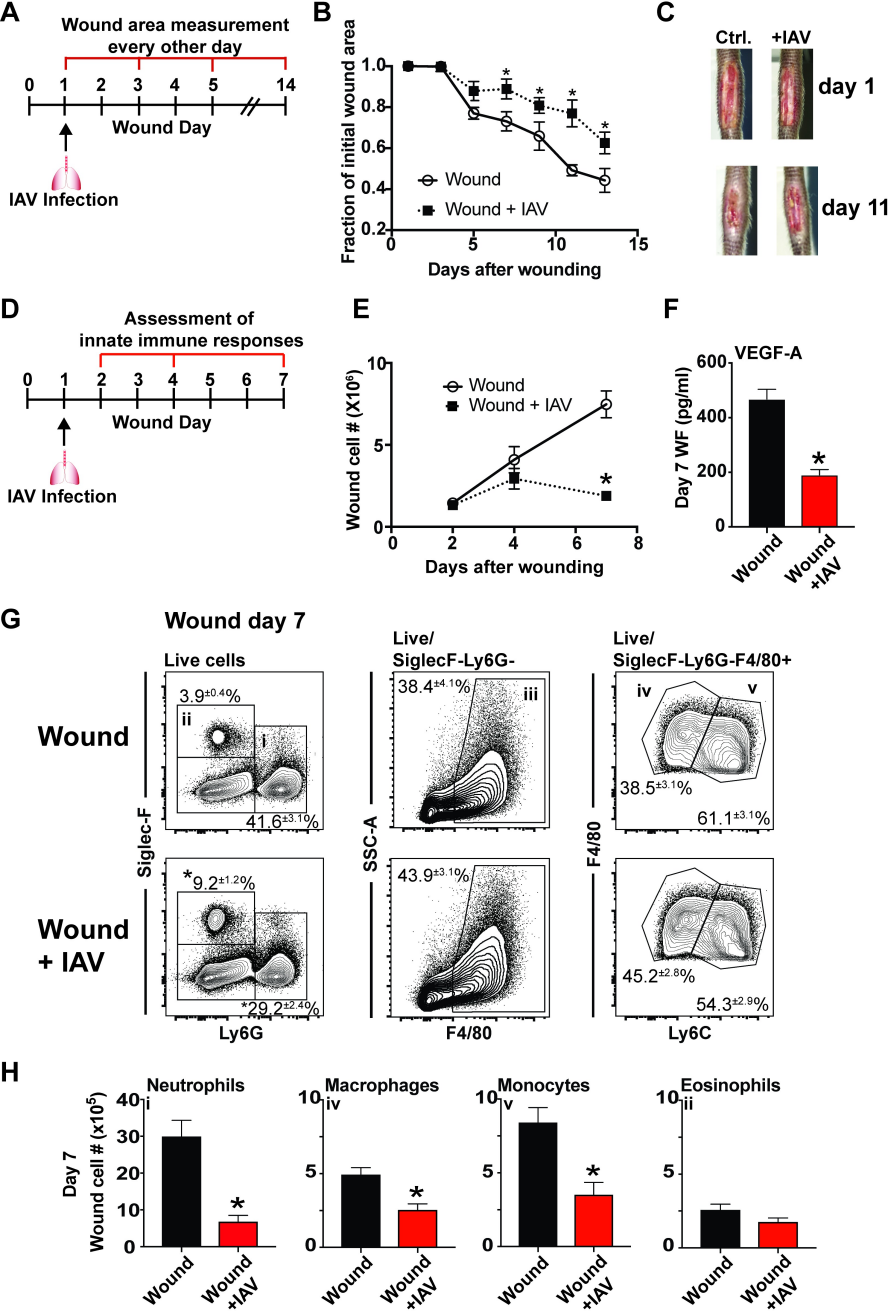
Cutaneous wound healing is delayed, and innate immune cell infiltration is impaired, in mice infected with IAV. Mice with excisional tail wounds were uninfected or infected intranasally with 500 PFU of IAV 24 hours after wounding. Wound area was measured for closure every other day for 14 days **(A)**. This is quantified in **(B**), and representative images of day 1 and day 11 wounds are shown in **(C)**. PVA sponges were subcutaneously implanted in mice that were uninfected or infected 24 hours later with IAV. Sponges were removed from cohorts of mice 2, 4, and 7 days after wounding **(D)**. Infiltrating cells were recovered from the sponges and quantified **(E).** Wound fluid from day 7 wounds was analyzed for levels of VEGFA **(F).** Immune cells from day 7 wounds were phenotyped by flow cytometry **(G)**. The total number of each cell type was determined at day 7 after wounding **(H)**. Data shown in B, E, F, and H are cumulative from at least 3 independent experiments (B n = 16 per group d1 and n = 12 per group for d3-13; E,F,H n = 12 per group). The mean values are displayed with SEM. Representative pictures from the data shown in C, and representative flow plots shown in G. For comparison of two groups the nonparametric Mann Whitney test was used. Results are considered statistically significant when the P value ≤ 0.05. Statistically significant changes between wound and wound +IAV are denoted by *.

To determine the effect of pulmonary IAV infection on the rate of wound healing, mice were subjected to excisional tail wounding and uninfected or infected with IAV 24 hours later (Fig 1A). In this model, a 1.0x0.3cm portion of skin is excised 0.5cm from the base of the tail. Murine tail skin wounds heal primarily by re-epithelization and provide a better model of human wound healing than dorsal skin punch biopsy wounds, which heal primarily by contraction [51]. The total wound area was measured every other day for a period of 14 days. The wounds from IAV-infected mice had delayed closure compared to those from control mice (Fig 1B and 1C). The initial healing rate was similar between the two groups, however by 7 days after wounding, the wounds from mice with IAV infection were significantly larger in area and healed at a slower rate from day 7 to day 11 (Fig 1B and 1C).

### Infection with IAV decreases innate immune cell infiltration into dermal wounds

In order to examine changes in the wound healing response at the cellular level, we used the PVA sponge implantation model. In this model PVA sponges are surgically implanted subcutaneously along the mouse dorsum. This wound model follows the inflammatory, angiogenic, and fibrotic stages of acute wound healing within the first two weeks of sponge implantation [24,25,29]. It allows for the retrieval of wound fluids and infiltrating leukocytes from implanted sponges by mechanical disruption. The sequence of inflammatory and repair responses mimics cutaneous wound models up to two weeks post-implantation, after which the non-resolving fibrotic response models a sterile foreign body reaction [18,19,23,25,31,52,53]. During the first seven days after sponge implantation, innate leukocytes, in particular monocytes and neutrophils, dominate the cellular wound healing response [18,19,23]. Wound day 7 is an inflection point representing the transition from the early inflammatory wound healing phase to the later reparative stages. At this time, inflammatory cytokines such as TNF-α and IL-6 are contracting in the wound, and pro-angiogenic and pro-fibrotic factors such as VEGF and TGF-β, are emerging [24,28].

For these studies, wounds were analyzed up to seven days after sponge implantation to assess the inflammatory phase of repair [19]. Wounded mice were uninfected or infected intranasally with IAV 24 hours after PVA sponge implantation. Half of the implanted sponges were removed at indicated time points (2, 4, and 7 days after wounding), and wound cells were collected for analysis (Fig 1D). The other half of the implanted sponges were used for wound fluid collection for analysis of cytokine and chemokine content in the healing wound environment. This enables a complete analysis of the early stages of the wound healing response mediated by innate immune cells. The cellularity of PVA sponge wounds in uninfected mice increased steadily over the first 7 days post-implantation. In mice that were infected with IAV 24 hours after wounding, the wound cellularity measured at days 2 and 4 after wounding was similar to uninfected control mice (Fig 1E). However, while the number of wound cells in uninfected mice approximately doubled between days 4 and 7 post-wounding, the number of wound cells in mice with concurrent infection did not increase. Correlating with the decrease in wound infiltrate, there was a greater than fifty percent decrease in the pro-angiogenic growth factor VEGF-A in the day 7 PVA sponge wound fluid (Fig 1F). VEGF-A is made locally in the wound primarily by repair macrophages and fibroblasts, and is essential in coordinating wound angiogenesis. In the PVA sponge wound model, it is expressed during the reparative stages of healing and is detectable in the wound fluid beginning around day 7 [19,54].

To determine whether the decrease in wound cellularity observed in IAV-infected mice at day 7 after wounding was attributable to changes in specific wound cell infiltrates, wound innate immune cell populations were analyzed by flow cytometry. The complete gating strategy for wound cells is shown in S1 Fig. On a percentage basis, there was a decrease in Ly6G^+^Siglec-F^−^cells, which are predominately neutrophils, in wounds from mice that had pulmonary IAV infection (Fig 1G). There was also a decrease in the percentage of Ly6C^high^F4/80^+^ monocytes and a relative increase in Ly6C^low^F4/80^+^ macrophages in mice with pulmonary viral infection (Fig 1G). The percentage of Siglec-F^+^Ly6G^–^ eosinophils also increased in mice with IAV infection (Fig 1G). However, when total cell numbers were calculated there was a significant decrease in all of these cell subsets at day 7 after wounding with the exception of eosinophils, which remained unchanged in number (Fig 1H). There was a larger decrease in the number of neutrophils and monocytes than in macrophages. Neutrophils and inflammatory monocytes traffic into wounds early, and the latter mature into macrophages over time in the PVA sponge wound environment [19]. This suggests that lung infection may block monocyte and neutrophil trafficking into the wound.

### Wounds from mice infected with IAV have decreased levels of chemokines and cytokines

**Fig 2:**
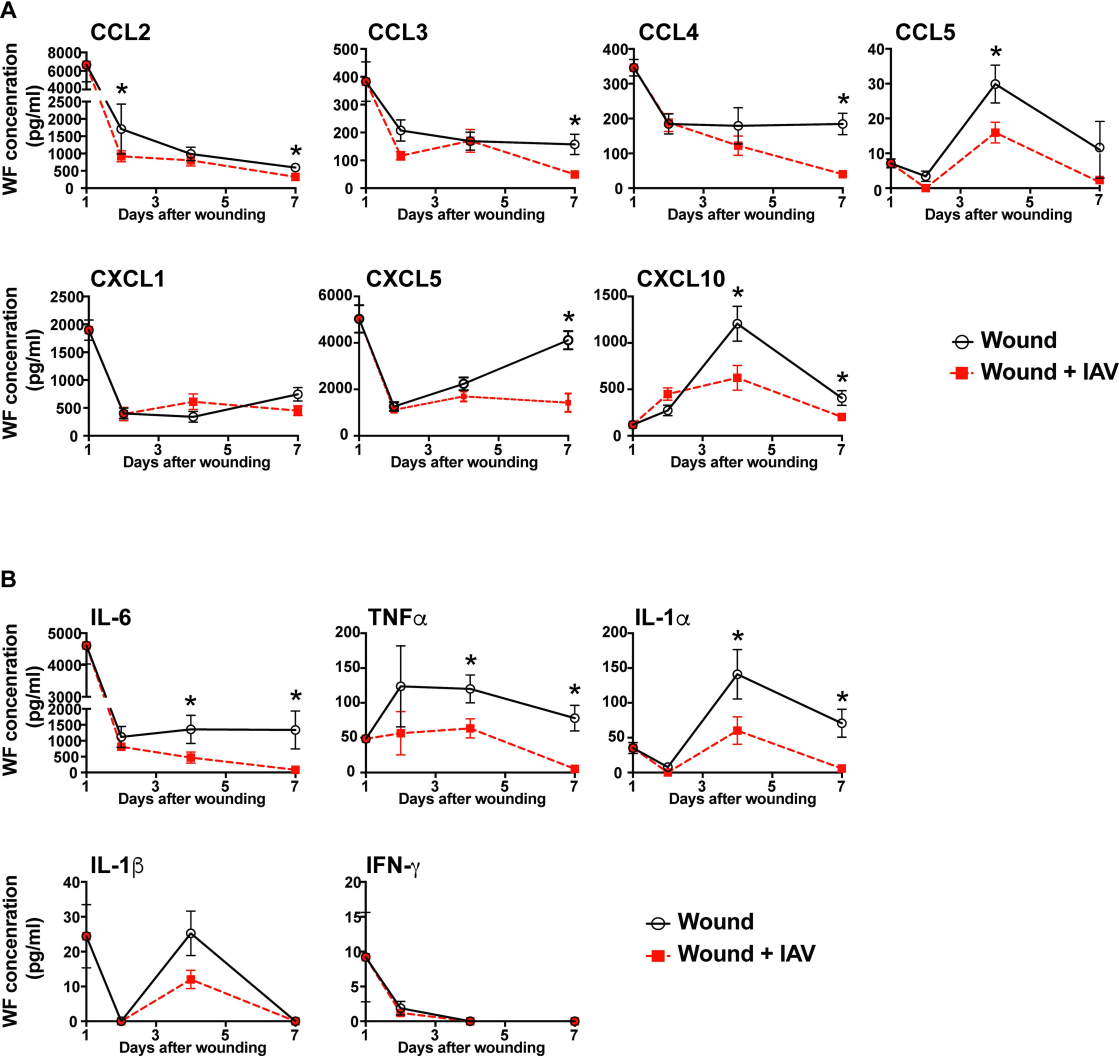
Chemokines and cytokines are decreased in wound fluids from mice with pulmonary IAV infection. Mice were wounded by subcutaneous PVA sponge implantation along the dorsum, and uninfected or infected with IAV 24 hours later. Wound fluid from uninfected control mice and mice with pulmonary IAV infection was examined for chemokine and cytokine levels. Chemokine levels were measured on days 1, 2, 4, and 7 after wounding **(A)**. Cytokine levels were measured at these same time points **(B)**. Data shown are cumulative from at least 3 independent experiments (All groups and time points have n = 12, except for d2 which has n = 13). The mean values are displayed with SEM. For comparison of two groups the nonparametric Mann Whitney test was used. Results are considered statistically significant when the P value ≤ 0.05. Statistically significant changes between wound and wound +IAV are denoted by *.

The absence of innate immune cell subsets in the wounds of IAV-infected mice suggested that the inflammatory environment that drives acute wound healing was disrupted [8, 36,53–55]. To assess this, we measured the levels of inflammatory cytokines and chemokines in the wounds of uninfected and infected mice. Soluble factors were extracted from sponges by centrifugation. An array of inflammatory factors involved in acute wound healing was assayed in the wound fluid including monocyte chemoattractants (CCL2, CCL3, CCL4, and CXCL10), neutrophil chemoattractants (CXCL1 and CXCL5), and cytokines (IL-1α, IL-1β, IL-6, IFN-α, IFN-γ, TNF-α, and GM-CSF). A number of these chemokines have overlapping functions in coordination of the innate immune response and angiogenic processes, which are critical for proper wound healing [55]. The chemokines CCL2, CCL3, CCL4, CCL5, CXCL1, CXCL5, and CXCL10, as well as the cytokines IL-6, TNF-α, IL-1α, IL-β, and IFN-γ were detectable in wound fluid within the first seven days post-wounding (Fig 2). Many inflammatory cytokines and chemokines were expressed at high levels one day after wounding and declined over time (Fig 2). CCL5, CXCL10, and IL-1α peaked at day 4 while CXCL5 and IL-1β had biphasic peaks (Fig 2). In mice that were infected with IAV, there were changes in cytokines and chemokine levels over the course of the healing wound. The chemokine CCL2 was decreased in wounds from mice infected with IAV as early as 2 days after wounding (Fig 2A). CXCL10 and CCL5 were decreased in wound fluids from IAV-infected mice 4 days after wounding (Fig 2A). This is remarkable as these decreases in chemokines and cytokines occurred before a significant change in innate immune cellularity, suggesting that the low cellularity could be partially due to a reduced chemotactic signal (Fig 1E). There was a decrease in the concentration of all detectable chemokines except CXCL1 in the day 7 wound fluids collected from IAV-infected mice, as compared to uninfected controls (Fig 2A). The proinflammatory cytokines IL-6 and TNF-α, as well as the alarmin IL-1α were all decreased in healing wounds at days 4 and 7 after wounding (Fig 2B). The overall modulation of cytokine and chemokine expression indicates that a lung infection can induce changes in the dermal wound environment.

### Wounding does not impact innate immune-mediated early control of pulmonary viral replication

**Fig 3:**
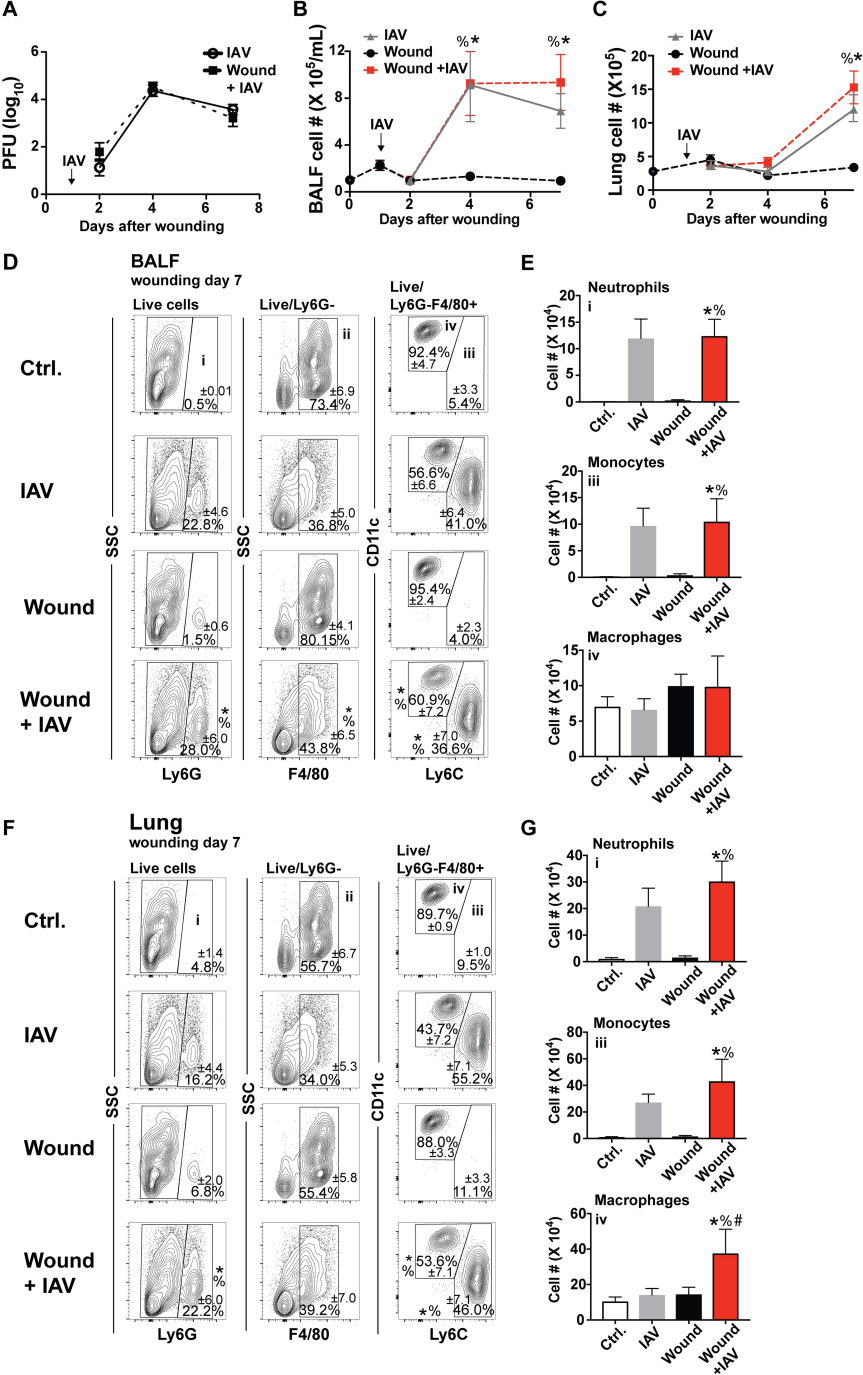
There is no increase in IAV load or cellularity of lungs of mice that are responding to dermal wounds and lung infection. Mice were wounded by subcutaneous PVA sponge implantation along the dorsum and uninfected or IAV-infected 24 hours later. Unwounded mice were either uninfected (control, wounding day 0) or infected with IAV. IAV load in the lung tissue of unwounded and infected or wounded and infected mice was quantified by viral plaque assay **(A)**. Immune cell infiltrate in the BALF **(B)** and processed lung tissue **(C)** was quantified from unwounded and uninfected mice (control, wounding day 0) or mice with IAV, wounds, or wounds and IAV by cell counting **(B)**. BALF was analyzed for innate immune cellular composition at day 7 by flow cytometry. Percentages are shown in **(D)**, and numbers in **(E)**. Innate immune cells from processed lung tissue were also quantified at day 7 by flow cytometry with percentages in **(F)** and numbers in **(G)**. Data shown are cumulative from at least 3 independent experiments (A n = 12 per group: B, C n = 9 for d1 and 4, and n = 20 for d0, 15 for d2, and 27 for d7; E, G Ctrl. n = 14, IAV n = 16, Wound n = 16, and wound+IAV n-17). In D and F representative flow plots are shown with the mean percentages from cumulative data displayed. The mean values are displayed with SEM. To compare 3 or more groups the Kruskal-Wallis one-way analysis of variance was used. Results are considered statistically significant when the P value ≤ 0.05. Statistically significant changes between control (wounding day 0) and wound + IAV are denoted by %, between IAV and wound +IAV are denoted by #, wound and wound +IAV are denoted by *.

Some studies have indicated that trauma suppresses the immune response in the lung [43]. Our PVA sponge model did not impact the initial control of IAV replication in the lung (Fig 3A). This could be because the PVA sponge wound is restricted to the subcutaneous space, does not involve blood loss, and therefore does not induce the high degree of trauma-induced immunosuppression that major surgery does. Wounding also did not impact the influx of immune cells into the lung, as wounded and unwounded mice responding to IAV infection had the same number of immune cells in the bronchoalveolar lavage fluid (BALF) (Fig 3B) and the lung parenchyma (Fig 3C). Since innate immune myeloid cells were decreased in the wounds of IAV infected mice, we investigated whether these same cells were also impacted 7 days after wounding in the lungs of mice that were responding to the two insults. The full gating strategy for BALF and lung cells can be seen in S2 Fig. To differentiate lung myeloid cell subsets, CD11c combined with F4/80 was used to define macrophages and monocytes. Neutrophils were identified by Ly6G expression. In the lung, alveolar macrophages express Siglec-F; therefore, this marker was not used to identify eosinophils in this compartment. Wounding did not alter the types of innate leukocytes, including Ly6G^+^ neutrophils and F4/80^+^Ly6C^hi^ monocytes, which infiltrated the BALF and lung in response to IAV infection 7 days after wounding (Fig 3D and 3E). However, in mice that were wounded and infected with IAV there was an increase in the number of CD11c^+^F4/80^+^Ly6C^-^ macrophages 7 days after wounding (Fig 3F and 3G), indicating a small change in the cellularity of the interstitial lung space.

### Wounding causes elevated levels of inflammatory cytokines and chemokines in the lung

**Fig 4:**
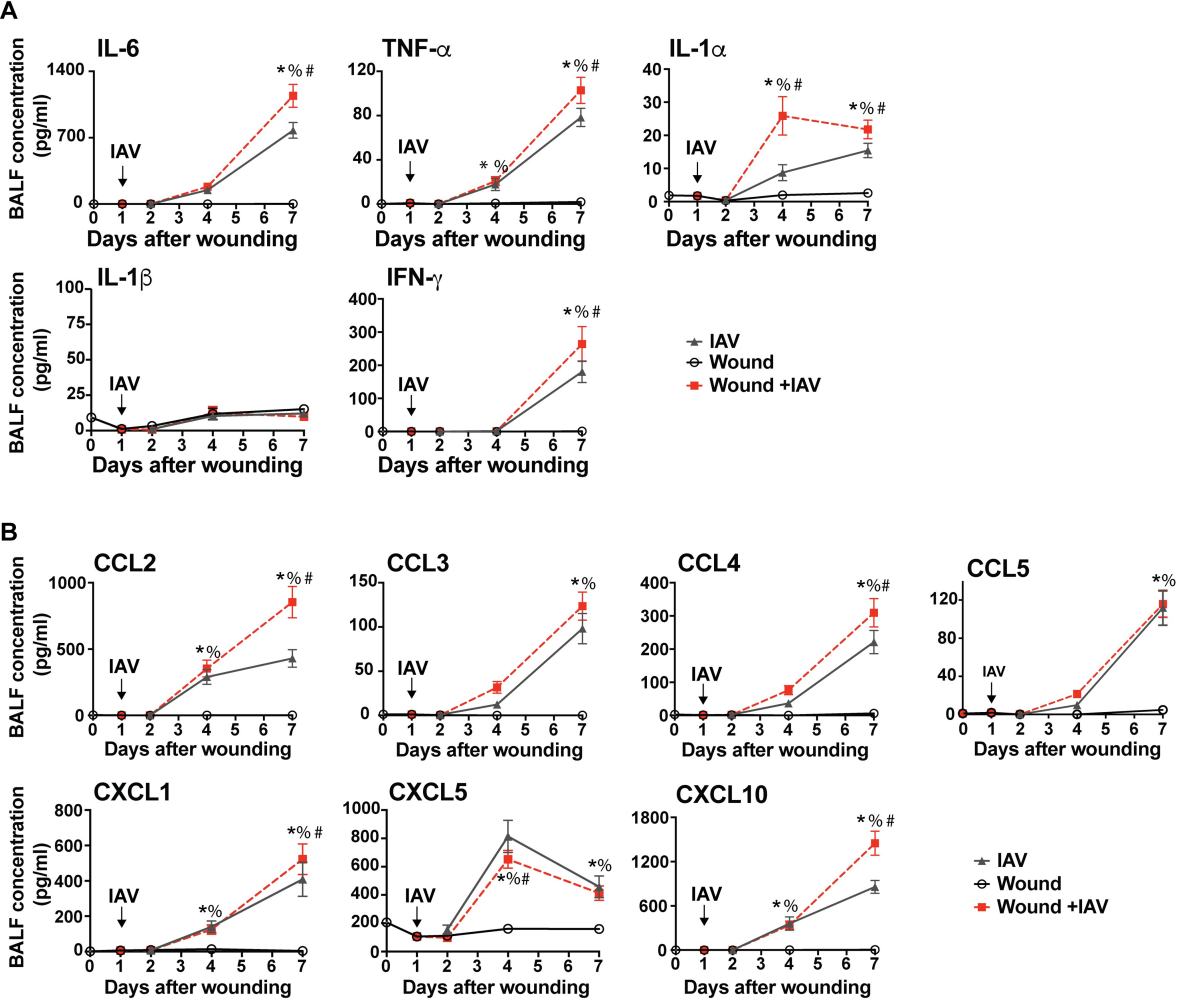
There is an increase in inflammatory cytokines and chemokines in the BALF of mice that are wounded and infected with IAV compared to wounding or IAV infection alone. Mice were wounded by PVA sponge implantation alone, infected with IAV alone, or wounded and IAV-infected 24 hours later. Control mice were unwounded and uninfected (wounding day 0). BALF levels of chemokines and cytokines were measured days 0, 1, 2, 4, and 7 after wounding. The inflammatory cytokines IL-6, TNFα, IL-1α, IL-β, and IFNγ were detected in the BALF **(A).** The chemokines CCL2, CCL3, CCL4, CCL5, CXCL1, CXCL5, and CXCL10 were detected in BALF **(B).** The data shown are cumulative from at least 3 independent experiments with n = 12 (on all groups except d7 where n = 18). The mean values are displayed with SEM. To compare 3 or more groups the Kruskal-Wallis one-way analysis of variance was used. Results are considered statistically significant when the P value ≤ 0.05. Statistically significant changes between control (wounding day 0) and wound + IAV are denoted by %, between IAV and wound +IAV are denoted by #, wound and wound +IAV are denoted by *.

The cellular innate immune response and the ability to clear IAV were not severely impacted in mice that were responding to the PVA sponge implantation wound. However, since cytokine and chemokine levels were impacted in the wounds prior to changes in cellularity, we examined these factors in the BALF from control, IAV-infected, wounded, and IAV-infected and wounded mice. Wounding alone did not significantly alter the levels of cytokines and chemokines in the BALF compared to unwounded and uninfected control mice (wounding day 0 in Fig 4). Infection with IAV caused a gradual increase in cytokine and chemokine concentrations as the cellular immune response increased in the lung. The cytokines IL-6, TNF-α, IL-1α, and IFN-γ were all increased in the BALF of mice that were responding to dermal wounds and infected with IAV, compared to mice infected with IAV alone (Fig 4A). Interestingly, the alarmin IL-1α was elevated in infected and wounded animals compared to infected mice alone as early as day 4 after wounding (Fig 4A). Responding to pulmonary IAV infection and dermal wounds simultaneously caused a decrease in the chemokine CXCL5 at day 4 after wounding, and an increase in CCL2, CCL4, CXCL1, and CXCL10 at day 7 after wounding, compared to IAV infection alone (Fig 4B). While there was a mild increase in cytokine- and chemokine-mediated inflammation in wounded and infected animals as compared to infected mice alone, this did not correlate with a loss of pulmonary vascular permeability, as assayed by total protein content in cell-free BALF (S3 Fig) [56–59].

### Infection with IAV increases systemic inflammation early after wounding

**Fig 5:**
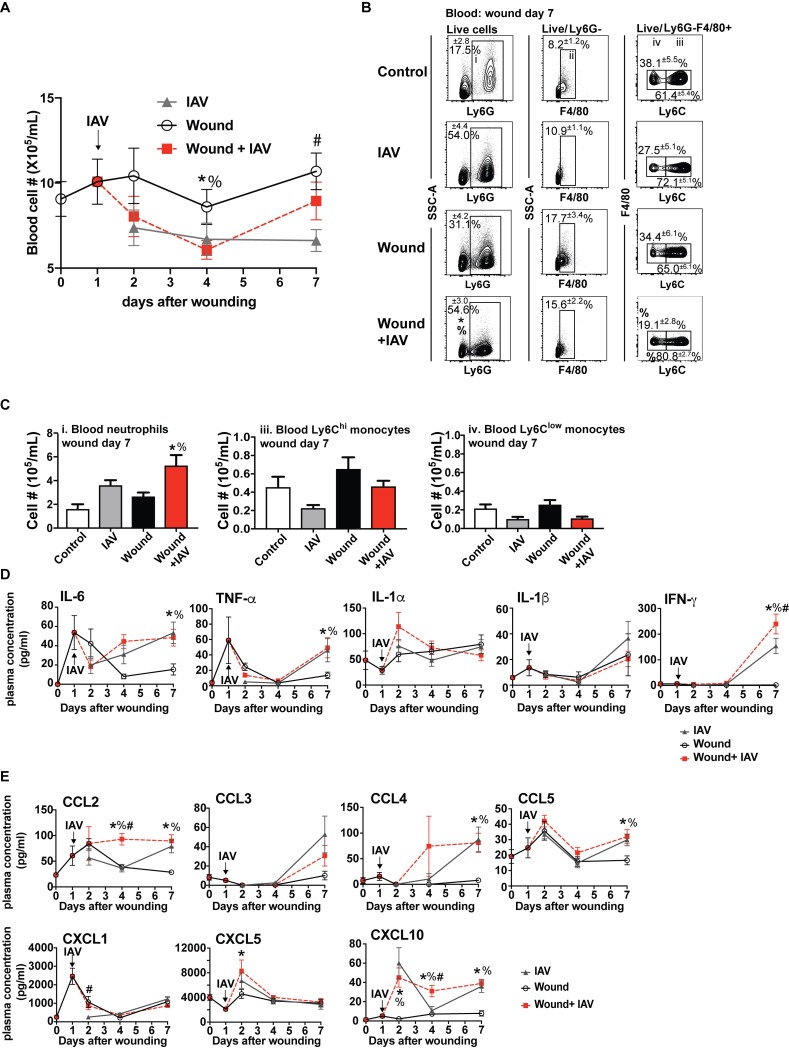
Wounding and IAV infection causes systemic inflammation. Mice were wounded by PVA sponge implantation and uninfected or infected with IAV 24 hours later. Control mice were unwounded and uninfected (wounding day 0). Cell numbers were counted in the blood at days 0, 1, 2, 4, and 7 after wounding with all 3 experimental groups (A). Flow cytometry analysis determined populations of innate immune cell subsets in the blood at day 7 after wounding (B). Flow cytometry analysis revealed changes in neutrophil and monocyte numbers on day 7 after wounding (C). Cytokine and chemokine levels were measured in the plasma on wound days 0, 1, 2, 4, and 7 from control mice or mice that were infected with IAV, wounded, or IAV infected and wounded (D+E). *p ≤ 0.05, wound+IAV vs. control; #p ≤ 0.05, wound+IAV vs. wound (E). The data shown are cumulative from at least 3 independent experiments (A: d0 n = 25 for all groups, d1 n = 9 for all groups, d2 n = 12 for all groups, d4 n = 16 for all groups, d7 n = 22 for all groups; B,C: n = 13 for control and n = 15 for remaining groups; D, E: n = 12 for all groups and 22 for day 7). The mean values are displayed with SEM. To compare 3 or more groups the Kruskal-Wallis one-way analysis of variance was used. Results are considered statistically significant when the P value ≤ 0.05. Statistically significant changes between control (wounding day 0) and wound + IAV are denoted by %, between IAV and wound +IAV are denoted by #, wound and wound +IAV are denoted by *.

We next examined the circulation to determine if local changes in the innate immune response to wounding and pulmonary pathogens reflected systemic changes in immune function. The cellularity of the blood was impacted primarily by infection, although there were distinct differences in wounded and infected animals (Fig 5A). PVA sponge wounding alone caused only slight modulations in blood cellularity over time. In contrast, as the innate immune system responded to IAV infection alone, there was a large concurrent decrease in the number of cells in the blood by day 2 (Fig 5A). Interestingly, the number of cells in the blood of mice that were infected and wounded initially dropped similarly to infected mice, but the recovery was quicker starting at day 4, and by day 7 the number of cells was close to that of uninfected wounded mice (Fig 5A).

The distribution of innate immune cell subsets in the blood was assessed by flow cytometry analysis (Fig 5B). The complete gating strategy for blood cells is shown in S4 Fig. Wounding and infection both caused an increase in the percentage of inflammatory cells including Ly6G^+^ neutrophils and F4/80^+^ monocytes in the blood compared to uninfected and unwounded control animals (wounding day 0) (Fig 5B). The most notable difference was that mice that were both infected and wounded had increased neutrophil numbers compared to wounded mice alone and control mice (wounding day 0) (Fig 5C).

Using the PVA sponge wound model, plasma from all four groups of mice was analyzed for cytokine and chemokine concentrations. Wounding caused an initial systemic spike of the inflammatory cytokines IL-6 and TNF-α at wound day 1 (Fig 5D). Plasma levels of IL-1β were slightly elevated later in the wound healing response, while IFN-γ was not impacted by the response to a healing wound (Fig 5D). The systemic cytokine and chemokine responses measured from days 4 to 7 in wounded and IAV-infected mice were similar to those measured in unwounded, IAV-infected mice. This suggests that the later systemic response in wounded and infected mice is dominated by the effects of pulmonary IAV infection. With the exception of IFN-γ, which was elevated at day 7 in the plasma of mice that had a wound and lung infection compared to IAV infection alone, the dual insults did not alter the profiles of the other cytokines (Fig 5D).

Mice that were infected with IAV after wounding also had elevated plasma chemokine concentrations (Fig 5E). In particular, CCL2, CXCL5, and CXCL10 had increased levels early after wounding and infection (Fig 5E). This augmentation of the response in the dual insult model was abrogated by day 7 after wounding (day 6 after infection), at which point the levels were close to that measured in mice infected with IAV alone (Fig 5E). This is in contrast to the changes in cytokine concentrations and blood cellularity, which were observed at later times during the response to infection and the healing wound. Overall these data indicate that there is a pattern of mild persistent systemic hyper-inflammation in mice that are responding to two insults.

## Discussion

The early innate immune response to both wound healing and infection share many properties [60]; we therefore hypothesized that the outcome of wound repair would be negatively influenced by the presence of an infection at a distal site. Previous observational studies by others demonstrated that both Sendai virus infection and murine hepatitis virus (MHV) infection altered skin wound tensile strength; however, the mechanisms for this altered wound healing were not elucidated [61]. To determine how a pulmonary infection with IAV impacts dermal wound healing we developed models of post-injury lung infection that allowed us to specifically examine the cellular responses of wound repair, as well as the rate of wound healing. As IAV is the most common viral cause of both community- and hospital-acquired pneumonia [62], this was an ideal model viral lung pathogen.

Acute wound healing is a coordinated process that occurs in three phases: inflammation, proliferation, and repair or fibrosis [2]. The initial inflammatory phase is critical to prevent wound infection, clear cell and tissue debris, and coordinate downstream angiogenic and fibrotic responses. Disruption of the inflammatory phase can result in impaired tissue repair responses and delayed healing. This is consistent with findings presented here, which demonstrate that a viral pulmonary infection negatively impacts wound cellularity, dampens wound cytokine and chemokine responses, and delays the rate of wound closure. Interestingly, these effects on the wound environment were uncoupled from the systemic response, in which we observed elevated concentrations of circulating chemokines and proinflammatory cytokines in mice responding to both injury and lung infection. This is distinct from the immune suppression observed in the systemic response to bacterial infection after pulmonary IAV infection [14]. In addition, the innate immune system was able to mediate early control of pulmonary IAV infection, as the viral load was not impacted in mice that had an ongoing wound healing response. Our results demonstrate that when the innate immune system is activated by consecutive challenges with contemporaneous responses, the response may be biased towards one particular site. Here we examined a coincident lung infection and a sterile injury of the skin, which demonstrated that the response was biased towards prioritizing the lung infection in detriment to the wound. This may be evidence for an immune triage mechanism offering the best chances of overall survival of the host.

The cells that were impacted the most by simultaneous responses to lung infection and wounding were neutrophils and monocytes/macrophages. These cells are important innate immune drivers of both acute wound healing and the early pulmonary antiviral response, which we hypothesized would affect their ability to respond to one or both of these successive and concurrent inflammatory insults. Prior studies with the PVA sponge model, as well as other wound models, have established that neutrophils are recruited to the injury site very early [2,3,18,19,24]. They are followed by Ly6C^hi^ inflammatory monocytes, and together they orchestrate the early inflammatory phase of wound repair. Monocytes mature over time into macrophages that contribute to wound repair responses such as angiogenesis and fibrosis [2, 19, 24, 53]. Our study shows that accumulation of neutrophils and monocytes is suppressed in PVA sponge wounds in mice with a lung infection. Work done by others has demonstrated that depleting monocytes/macrophages at early stages of wound repair negatively impacts healing [8,53]. Interestingly, the decrease in the proportion of inflammatory monocytes in the wounds of infected mice was accompanied by a relative increase in the proportion of wound macrophages, which could suggest an accelerated transition of monocytes to wound repair macrophages. When examining absolute numbers, however, both populations were reduced, suggesting that the loss of infiltrating monocytes may instead lead to a reduced number of monocyte-derived macrophages recovered from the wounds of infected mice [19].

In addition to decreased inflammatory cells, the decrease of cytokines such as IL-6 has important implications in the wound healing process [26, 63–66]. Our data from studies using the PVA sponge model demonstrated suppressed acute wound cellular and cytokine responses including IL-6 in the presence of a simultaneous lung infection. IL-6 has been shown to control levels of adhesion molecules, growth factors, and chemokines that are essential to the healing wound [29,30,67]. Additionally, the production of the angiogenic growth factor, VEGF-A, was also suppressed in mice with concurrent pulmonary infection suggesting downstream consequences for the initiation of the repair stage of wound healing.

Coordinated chemokine expression is essential to orchestrating the various phases of wound repair. We measured decreased expression of CCL2, CCL5, and CXCL5 at early, mid, and late acute stages of healing, respectively, in the wound fluids of IAV-infected mice. These chemokines recruit innate immune populations such as neutrophils and inflammatory monocytes, and are pro-angiogenic, suggesting that their sequential disruption may inhibit the progression through the early stages of wound repair. CXCL10 levels were also reduced in the wound fluids of IAV-infected mice at 4 days post-wounding. In the wound, CXCL10 is an angiostatic signal that promotes the transition from granulation to resolution phases, further suggesting that dysregulated chemokine signaling negatively impacts wound healing.

CXCL10 is normally induced by IFN-γ, however examination of local and systemic IFN-γ production suggested that wound CXCL10 was induced independently. For example, wound fluid IFN-γ levels were very low at all time points examined, measuring only 10 pg/mL at its peak one day after wounding, and this IFN-γ peak preceded the height of CXCL10 expression in the wound fluid by three days. Furthermore, there were no differences in the wound fluid concentration of IFN-γ between uninfected mice and mice with IAV infection, whereas CXCL10 expression was diminished in IAV-infected mice. A recent study showed that CXCL10 can be induced independently of the IFN response [68]. In particular this has been shown in response to viruses, although it has yet to be explored, to our knowledge, in wound healing responses. Some studies have demonstrated the ability of oral keratinocytes and fibroblasts to produce CXCL10 transcripts *in vitro* in response to TNF-α, IL-1α, and IL-4 stimulation. Keratinocytes and fibroblasts also have roles in healing cutaneous wounds, suggesting that IFN-γ-independent CXCL10 expression could occur in the wound [69–71]. In addition, impaired chemokine levels may stem from numerical deficits or functional defects in their numerous cellular sources, such as platelets, endothelial cells, and leukocytes. Systemic IFN-γ could also induce local wound production of CXCL10. IFN-γ was detected in the plasma, but its systemic peak succeeded the peak of both wound and plasma CXCL10, suggesting that, in general, the modulation of CXCL10 expression in IAV-infected mice was independent of IFN-γ signaling. Interestingly, plasma CXCL10 was induced by IAV infection more rapidly than in the BALF, suggesting an extrapulmonary site of production, such as the liver, which has been shown to become activated during influenza infection [14, 72–73].

In contrast to what was observed in the healing wound, there was a mild increase in systemic inflammation in mice with both wounds and infection. IAV infection with and without wounding resulted in a drop in circulating leukocytes 4 days after wounding and 3 days after IAV infection, likely due to extravasation to the infected lung. At 7 days after wounding, wounded mice had the highest number of circulating leukocytes while blood cellularity was lowest in IAV-infected mice. Mice with wounds and infection had intermediate levels of blood cellularity, suggesting that circulating cells were influenced by both inflammatory sites. This trend was also reflected in circulating neutrophils and monocytes. There were also increased concentrations of systemic cytokines and chemokines measured in the plasma of wounded and IAV-infected mice. The trends in plasma cytokine content reflected those measured in the BALF at 7 days post-wounding, suggesting that IAV infection was the major driver of systemic inflammation in wounded and infected mice at later time points. At day 4 after wounding there was an increase in the chemokines CCL2 and CXCL10 in the serum from mice that had been both wounded and infected. Both CXCL10 and CCL2 are important in the recruitment of many cell types, including monocytes, to inflamed tissue. These synergistic inflammatory cytokine and chemokine responses may suggest that the presence of dual inflammatory insults signals to increase leukocyte mobilization from sites of hematopoiesis to the circulation for increased availability to the inflamed periphery.

There was also an increase in markers of inflammation in the infected lungs of mice with PVA sponge wounds. As observed in the plasma, BALF recovered from the lungs of wounded and infected mice had increased levels of the chemokines CCL2 and CXCL10. BALF concentrations of IL-6, TNF-α, IL-1α, and IFN-γ were also increased in the BALF of wounded and infected mice when compared to IAV-infected mice alone. Macrophage numbers were slightly elevated in the lung and could be responsible for this mild inflammatory response. Alternatively, this could be due to increased cytokine production on a per-cell basis. Activated non-immune cells, such as the epithelium or endothelium, may also contribute to increased inflammation in the lung. Together these data suggest that, while the innate immune response is able to control the early stages of viral infection, it appears that distal wounding causes dysregulation of certain aspects of pulmonary inflammatory immune responses during IAV infection. However, this increase in pulmonary inflammation in wounded mice does not appear to contribute to additional lung damage compared to IAV-infected mice without wounds.

Our study shows that when confronted with both a pulmonary infection and a dermal wound the immune response is impacted in all compartments; however, the impact on the wound healing response is the greatest. The data presented here suggest that a distal infection disrupts the inflammatory phase of wound repair, resulting in delayed healing. Surprisingly the innate immune mediated control of the lung infection was not impacted by the injury. This study specifically addresses the effect that lung infection has on cutaneous and subcutaneous wounds, but it could also impact the healing of internal injuries. In the patient population, the complications that arise from the immune response combating simultaneous inflammatory events have important consequences. Altered wound healing could increase susceptibility to further complications leading to increased patient morbidity [17,36,37]. This proof of concept study opens a new area of research that aims to understand the intricacies underlying the innate immune response in complex biological systems facing multiple inflammatory insults. How the immune response is directed towards the lung at the expense of the healing wound and how this prioritization can be altered are important areas of future study. Given the numerous important functions of the innate immune response, these results have implications for many diseases, which remain to be explored. The ultimate aspiration is to advance the clinical outcomes of patients with complex disease sequelae.

## Materials and methods

### Ethics statement

All animal studies were approved by the Brown University Institutional Animal Care and Use Committee and carried out in accordance with the Guide for the Care and Use of Animals of the National Institutes of Health. Brown University adheres to the “U.S. Government Principles for the Utilization and Care of Vertebrate Animals Used in Testing, Research, and Training”, “PHS Policy on Humane Care and Use of Laboratory Animals”, “USDA: Animal Welfare Act & Regulations”, and “the Guide for the Care and Use of Laboratory Animals”. The University is accredited by the Association for Assessment and Accreditation of Laboratory Animal Care International (AAALAC). Brown University’s PHS Assurance Number: A3284-01, expiration date: July 1, 2018. The USDA Registration Number is 15-R-0003. Brown University IACUC was approved on October 8, 2013, and the animal protocol number is 1308000011. The de novo renewal was approved on September 28,2016 and the animal protocol number is 1608000222.

### Study design

Group sizes of studies were determined by power analysis. To have confidence in our data we aimed to have a p value (alpha) of .05, and a power of .80 (beta of 20%) in a 1-way ANOVA. All power analysis was done using Power Analysis in R package: pwr.anova.test(k = 4, f = .25, sig.level = .05, power = .8 n = 4). Means and shared sigma values (standard deviation) were used from biological data generated in preliminary experiments, and from published work from lung infections. Exclusion of mice from data collection was determined based on extensive experience with murine pulmonary infection and sterile wound models. No mice were excluded from these studies according to the following criteria: 1) mice were not infected as determined by observation of physical appearance and measurement of viral titers and lung cellularity and 2) mice displayed overt wound infection, indicated by swelling, redness, pus, and irritation of the surrounding skin. Study endpoints were determined prospectively based on prior experience with mouse models of pulmonary infection and sterile wound healing. Each experiment was repeated a minimum of three times, with a minimum of three mice per group.

### Mice

C57BL/6J mice were purchased from The Jackson Laboratory. As is consistent with previously published work with the wound model and to prevent gender specific complications, male mice 8–12 weeks of age were used in all experiments [74,75].

### Pulmonary infection

Mice under anesthesia and analgesia by ketamine (60–80 mg/kg) and xylazine (30–40 mg/kg) injection were administered IAV intranasally in a volume of 30 μL using a sterile saline vehicle. Mice were infected with 500 PFU influenza A virus (A/WSN/33 (H1N1)) strain. Influenza A virus was obtained from Akiko Iwasaki at Yale University. It was propagated using MDCK cells using standard procedures as described [12]. Mice were monitored daily for a minimum of three days, and every other day for the remainder of the experiment.

### Polyvinyl alcohol sponge implantation

Polyvinyl alcohol (PVA) sponge implantation surgeries were performed under anesthesia and analgesia by ketamine (60–80 mg/kg) and xylazine (30–40 mg/kg) injection. Backs were shaved and cleaned with povidone-iodine solution and isopropyl alcohol. Six 1cm×1 cm×0.3 cm sterile PVA sponges (Ivalon, PVA Unlimited, Inc.) were placed into subcutaneous pockets through a 2cm midline dorsal incision under sterile conditions. The incision was closed with surgical clips. Mice were monitored daily for the first three days following surgery then a minimum of every other day for the remainder of the duration of the experiment.

### Full-thickness tail wounding

The tail was cleaned with povidone-iodine solution and isopropyl alcohol. A 1cm x 0.3cm area of the skin was excised using a scalpel 0.5cm from the base of the tail. The wound bed was covered with a spray barrier film (Cavilon, 3M). Wound area was measured using calipers. Length and width measurements were taken at the midpoints of the wound bed. Tail wound images were acquired from a fixed position using a 12-megapixel iSight camera and were analyzed using ImageJ (NIH). All measurements were done in a blinded fashion to prevent bias.

### Wound fluid and cell isolation

Mice were euthanized by CO_2_ asphyxiation prior to sponge removal. For wound fluid collection, the three sponges implanted left of the midline were removed and placed in the barrel of a 5mL syringe, which was placed in a tube and centrifuged for fluid collection. The three remaining sponges that were implanted right of the midline were removed from each animal and placed in 1x HBSS medium (1% FCS/penicillin/streptomycin/1M hepes), and the cells were isolated using a Stomacher (Tekmar). Wound cells were washed with 1x HBSS medium and red blood cells were lysed. Cell counts were obtained using a Moxi Z Automated Cell Counter (Orflo).

### Plasma and blood cell collection

Blood was collected retroorbitally into heparinized tubes at indicated time points. Each collection time point represents an independent sample group. Plasma, leukocytes, and red blood cells were fractionated by centrifugation in Wintrobe Tubes (CMSLabcraft). Plasma was collected for cytokine analyses. The buffy coat layer containing leukocytes was collected into a fresh tube and residual red blood cells removed by water lysis. Cells were counted with a Moxi Z Automated Cell Counter (Orflo) and used for flow cytometry analyses. The remaining red blood cell layer was discarded.

### BALF collection

To collect bronchoalveolar lavage fluid (BALF), the trachea was exposed, and a BD Venflon IV catheter was inserted into the trachea. The needle was removed and a 1ml syringe filled with PBS was inserted. The lung was rinsed with 1ml PBS twice using an attached syringe. Cell-free supernatants were collected for cytokine analyses and protein content quantification. Cells were counted with a Moxi Z Automated Cell Counter (Orflo) and used in flow cytometry analyses.

### BALF total protein content quantification

The concentration of protein in the BALF was determined using the bicinchoninic acid (BCA) assay according to the manufacturer’s instructions (Pierce Chemical Co.). A dilution series was tested for each sample against an Albumin standard.

### Lung tissue cell collection

For isolation of cells from lungs, the right superior and middle lobes were perfused with 20 ml of PBS. The lung tissue was cut into small pieces and incubated for 45min at 37 degrees C in 4ml of media containing type 4 collagenase (Worthington Biochemical Corporation) and DNAse I (Sigma-Aldrich). Afterwards, digested lung tissue was passed through a 70uM cell strainer to make a single cell suspension. After centrifugation the cell pellet was re-suspended in 4ml of 40% Percoll/RPMI and carefully layered over 4ml of 80% Percoll/PBS. The gradient was centrifuged at room temperature for 20 minutes at 600g with minimal acceleration and deceleration. Cells assembled in the interphase were collected, and washed with 10ml RPMI media containing 5% fetal calf serum by centrifugation.

### Viral titration

Viral PFU were obtained using the unperfused right inferior lobe. The lobe was homogenized in 1 ml of PBS using the Polytron 2100 homogenizer. Viral plaque forming units (PFUs) were obtained by titration of diluted supernatant on MDCK cells as described elsewhere [14]. Briefly, cell homogenate was diluted in PBS and 100 μl was plated on MDCK cells in 6 well plates. After a 1 hour incubation at 37° C the virus was removed and the cells were covered in 50% media/50% oxoid agar supplemented with DEAE dextran, NaHCO_3_, and penicillin/streptomycin. After 3 days the agar was removed, the plates were stained with crystal violet, and viral plaques were counted.

### Flow cytometry analysis of cell subsets

The following antibodies were used to identify cell subsets: Ly6C-FITC (AL-21, BD Biosciences), F4/80-PerCP-Cy5.5 or APC eFluor660 (BM8, eBioscience), Siglec-F-PE (E50-2440, BD Biosciences), CD11c-PE or BV711 (HL3, Biolegend), and Ly6G-PerCP eFluor710 or V450 (1A8, eBioscience or BD Biosciences). Dead cells were excluded from analyses using Fixable Viability Dye APC eFluor780 or BV506 (eBioscience).

Surface staining: Cells from all samples were adjusted to an equal concentration, and treated with anti-CD16/CD32 Fc receptor blocking antibody (clone 2.4G2) in 1x PBS (1% FBS) for 10 minutes on ice. Surface staining antibodies were then added and incubated for 15 minutes on ice. Cells were washed with 1x PBS then incubated with Fixable Viability Dye diluted in 1x PBS for 15 minutes on ice. Cells were washed, then fixed with 1% paraformaldehyde for 15 minutes on ice.

Samples were acquired using an Attune NxT Acoustic Focusing Cytometer with Attune Software or a BD FACSAria with FACSDiva Software. Analyses were performed using FlowJo v10 software (Tree Star, Inc.). Gate placement was determined using isotype, FMO, or unstained control samples. Total cell numbers of each cell subset was obtained by using the total cell counts of the compartment as described above, and multiplying by the percent of total viable cells as determined by flow cytometry.

### Cytokine analysis

Cytokine concentrations were determined in wound fluid, BALF, and plasma using a custom LEGENDplex bead-based immunoassay (BioLegend) according to manufacturer instructions, except for VEGF-A, CXCL1, and CXCL5. The concentrations of these cytokines were determined using DuoSet sandwich ELISA kits (R&D Systems) according to manufacturer instructions.

### Statistical analysis

Biostatistical analysis was carried out using the GraphPad Prism software package. For comparison of two groups the nonparametric Mann Whitney test was used. To compare 3 or more groups the Kruskal-Wallis one-way analysis of variance was used, with Tukey-Kramer test for post-hoc analysis. All the groups were compared to each other. However, for clarity, the statistics shown are the most relevant for this study. This means a comparison of Wound + IAV with control, Wound, or IAV at all time points. Results are considered statistically significant when the P value ≤ 0.05. Statistically significant changes between control and wound + IAV are denoted by %, between IAV and wound +IAV are denoted by #, wound and wound +IAV are denoted by *.

## Supporting information

S1 Fig Gating strategy for cells isolated from PVA sponge wounds. Doublets were excluded using FSC-A, FSC-H, SSC-A, and SSC-H (i and ii). Dead cells were excluded using a fixable viability dye (iii). Leukocytes were gated on using FSC and SSC parameters (iv). Eosinophils (SiglecF+, Ly6G-) and neutrophils (SiglecF^-^Ly6G^+^) were identified using the marker Siglec-F and Ly6G (v and vi). Ly6G^–^Siglec-F^−^cells (vii) were gated on F4/80^+^ to identify macrophages and monocytes (viii). Macrophages (ix) and monocytes (x) were distinguished based on expression of Ly6C.
(TIF)

S2 Fig Gating strategy for cells isolated from lung and BALF. Doublets were excluded using FSC-A, FSC-H, SSC-A, and SSC-H (i and ii). Dead cells were excluded using a fixable viability dye (iii). Leukocytes were gated on using FSC and SSC parameters (iv). Neutrophils (Ly6G^+^) were identified using the marker Ly6G (v). Ly6G^–^ (vi) cells were gated on F4/80^+^ cells to identify macrophages and monocytes (vii). Macrophages (viii) and monocytes (ix) were distinguished based on expression of Ly6C and CD11c.
(TIF)

S3 Fig Protein content in the BALF. To determine lung damage total protein content in the BALF was measured.
For comparison of two groups the nonparametric Mann Whitney test was used. To compare 3 or more groups the Kruskal-Wallis one-way analysis of variance was used. Results are considered statistically significant when the P value ≤ 0.05. Statistically significant changes between control and wound + IAV are denoted by %, between IAV and wound +IAV are denoted by #, wound and wound +IAV are denoted by *.
(TIF)

S4 Fig Gating strategy for cells isolated from the blood. Doublets were excluded using FSC-A, FSC-H, SSC-A, and SSC-H (i and ii). Dead cells were excluded using a fixable viability dye (iii). Leukocytes were gated on using FSC and SSC parameters (iv). Eosinophils (SiglecF^+^) were excluded using SSC and Siglec-F (v). Neutrophils (Ly6G^+^) were identified using the marker Ly6G (vi). Ly6G^–^ cells (vii) were gated on F4/80^+^ cells to identify macrophages and monocytes (viii). Monocytes were separated into subsets based on expression of Ly6C (ix and x).
(TIF)
