# Supplementary figures and images for: Pulmonary influenza A virus infection leads to suppression of the innate immune response to dermal injury
Source: PLoS Pathog. 2018 Aug 23;14(8):e1007212. doi: 10.1371/journal.ppat.1007212 (PMC6107272; doi:10.1371/journal.ppat.1007212)

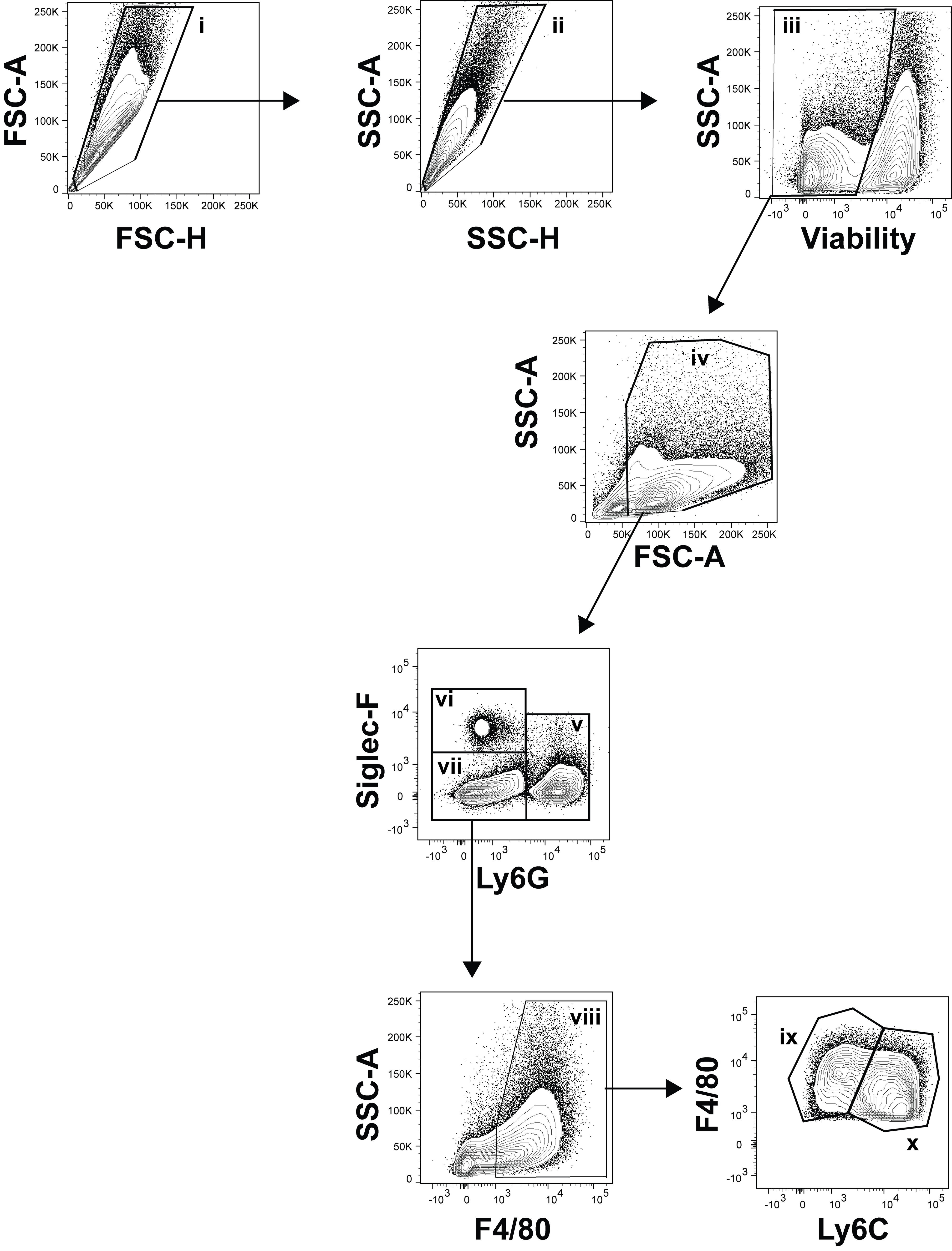

Supplement: S1 Fig — Doublets were excluded using FSC-A, FSC-H, SSC-A, and SSC-H (i and ii). Dead cells were excluded using a fixable viability dye (iii). Leukocytes were gated on using FSC and SSC parameters (iv). Eosinophils (SiglecF+, Ly6G-) and neutrophils (SiglecF-Ly6G+) were identified using the marker Siglec-F and Ly6G (v and vi). Ly6G–Siglec-F−cells (vii) were gated on F4/80+ to identify macrophages and monocytes (viii). Macrophages (ix) and monocytes (x) were distinguished based on expression of Ly6C. (TIF) [file ppat.1007212.s001.tif]

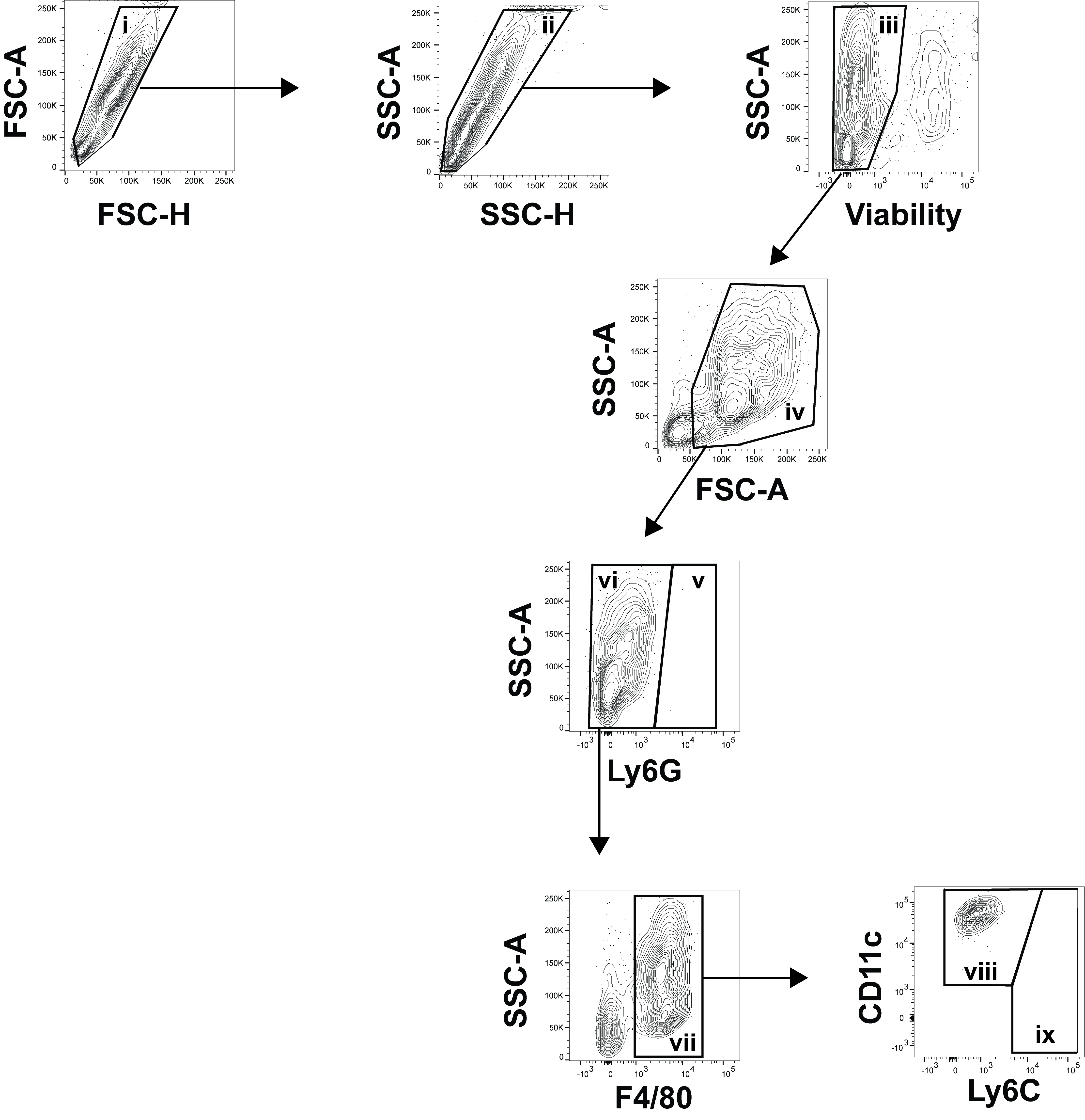

Supplement: S2 Fig — Doublets were excluded using FSC-A, FSC-H, SSC-A, and SSC-H (i and ii). Dead cells were excluded using a fixable viability dye (iii). Leukocytes were gated on using FSC and SSC parameters (iv). Neutrophils (Ly6G+) were identified using the marker Ly6G (v). Ly6G– (vi) cells were gated on F4/80+ cells to identify macrophages and monocytes (vii). Macrophages (viii) and monocytes (ix) were distinguished based on expression of Ly6C and CD11c. (TIF) [file ppat.1007212.s002.tif]

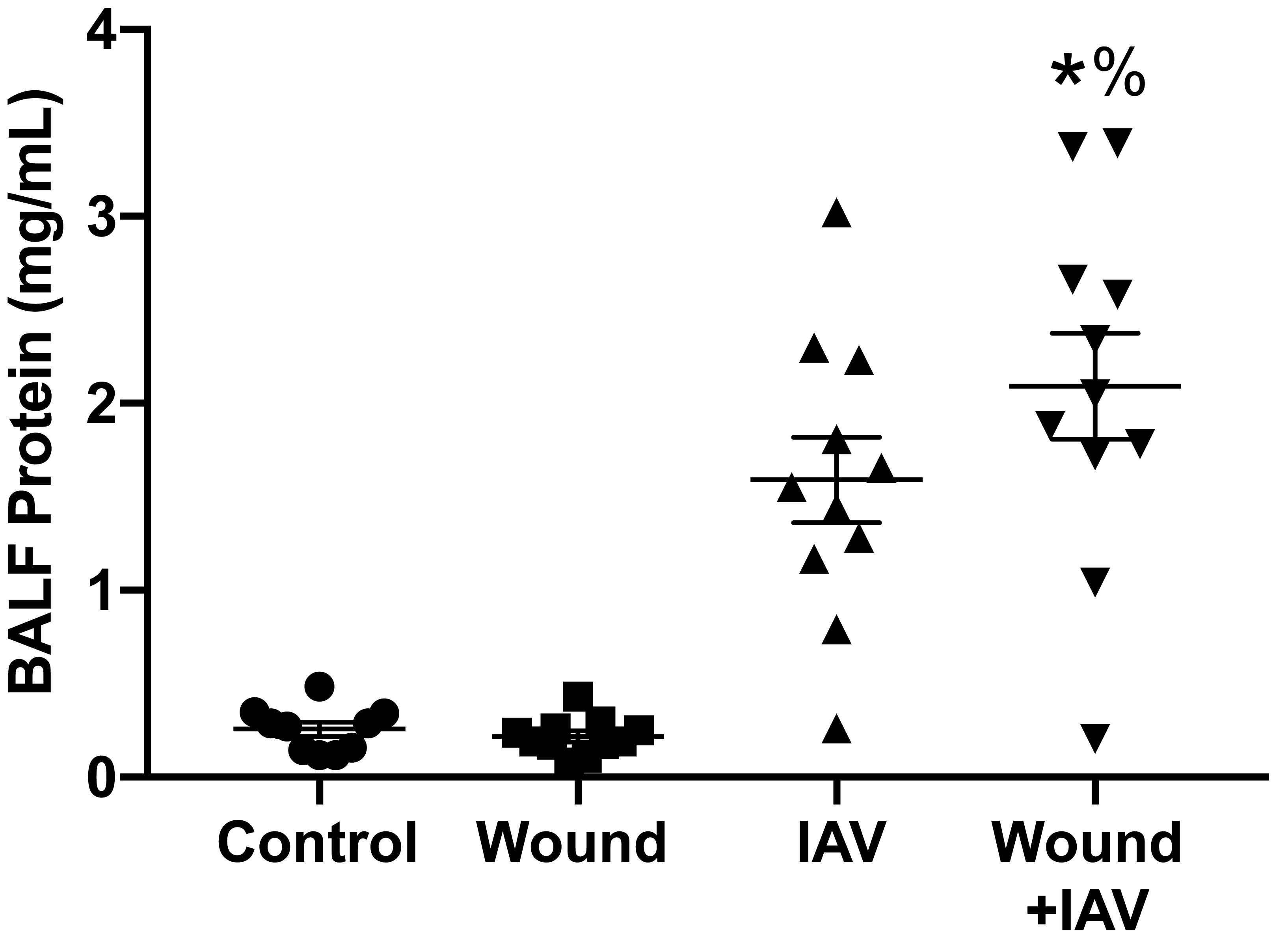

Supplement: S3 Fig — To determine lung damage total protein content in the BALF was measured. For comparison of two groups the nonparametric Mann Whitney test was used. To compare 3 or more groups the Kruskal-Wallis one-way analysis of variance was used. Results are considered statistically significant when the P value ≤ 0.05. Statistically significant changes between control and wound + IAV are denoted by %, between IAV and wound +IAV are denoted by #, wound and wound +IAV are denoted by *. (TIF) [file ppat.1007212.s003.tif]

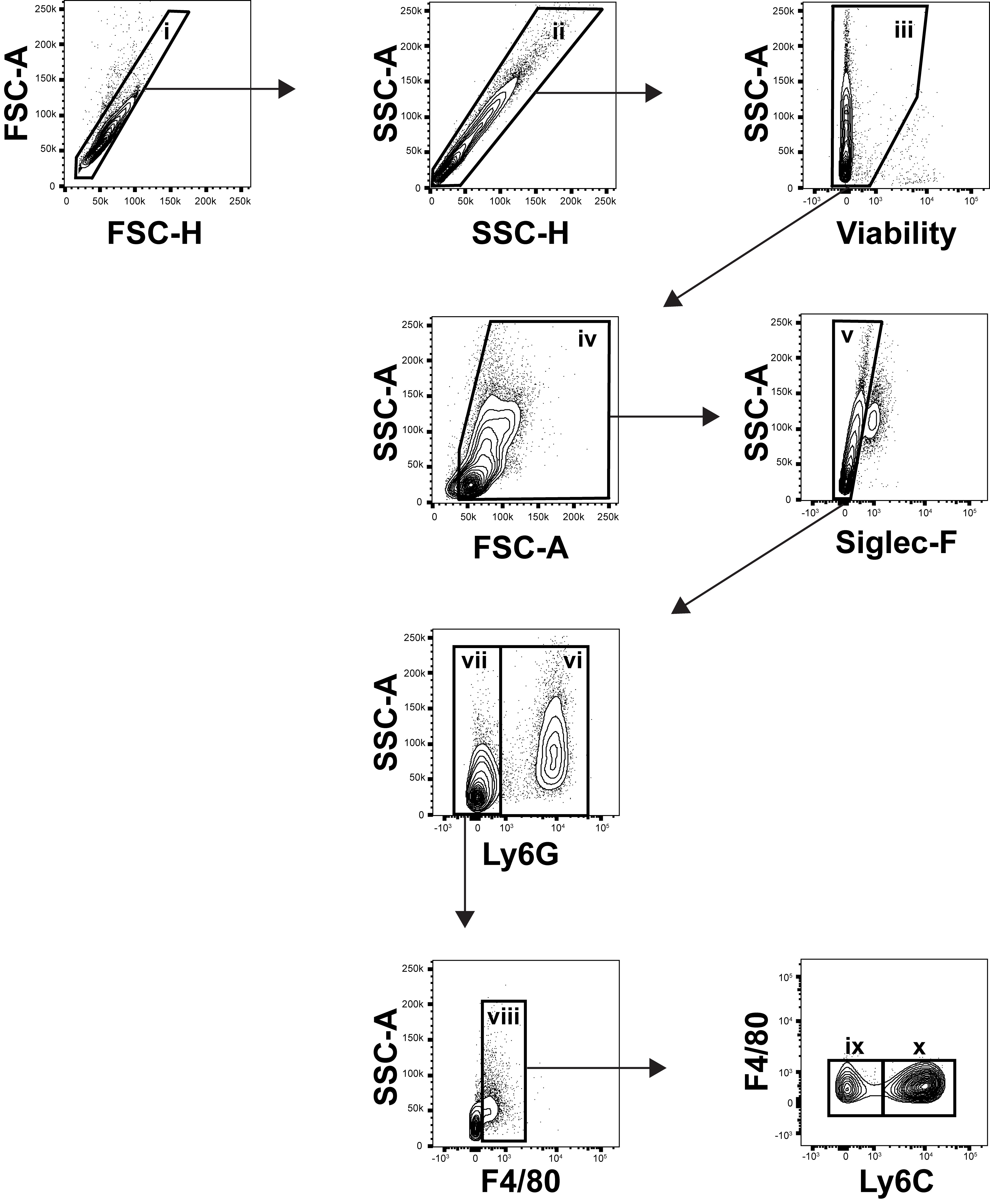

Supplement: S4 Fig — Doublets were excluded using FSC-A, FSC-H, SSC-A, and SSC-H (i and ii). Dead cells were excluded using a fixable viability dye (iii). Leukocytes were gated on using FSC and SSC parameters (iv). Eosinophils (SiglecF+) were excluded using SSC and Siglec-F (v). Neutrophils (Ly6G+) were identified using the marker Ly6G (vi). Ly6G– cells (vii) were gated on F4/80+ cells to identify macrophages and monocytes (viii). Monocytes were separated into subsets based on expression of Ly6C (ix and x). (TIF) [file ppat.1007212.s004.tif]
